# Cycad-Weevil Pollination Symbiosis Is Characterized by Rapidly Evolving and Highly Specific Plant-Insect Chemical Communication

**DOI:** 10.3389/fpls.2021.639368

**Published:** 2021-04-30

**Authors:** Shayla Salzman, Damon Crook, Michael Calonje, Dennis W. Stevenson, Naomi E. Pierce, Robin Hopkins

**Affiliations:** ^1^Plant Sciences, Cornell University, Ithaca, NY, United States; ^2^Organismic and Evolutionary Biology, Harvard University, Cambridge, MA, United States; ^3^Otis Laboratory, USDA-APHIS-PPQ CPHST, Otis ANGB, MA, United States; ^4^Montgomery Botanical Center, Coral Gables, FL, United States; ^5^New York Botanical Garden, Bronx, NY, United States

**Keywords:** coevolution, brood-site mutualism, chemical signaling, Coleoptera, Caribbean

## Abstract

Coevolution between plants and insects is thought to be responsible for generating biodiversity. Extensive research has focused largely on antagonistic herbivorous relationships, but mutualistic pollination systems also likely contribute to diversification. Here we describe an example of chemically-mediated mutualistic species interactions affecting trait evolution and lineage diversification. We show that volatile compounds produced by closely related species of *Zamia* cycads are more strikingly different from each other than are other phenotypic characters, and that two distantly related pollinating weevil species have specialized responses only to volatiles from their specific host *Zamia* species. Plant transcriptomes show that approximately a fifth of genes related to volatile production are evolving under positive selection, but we find no differences in the relative proportion of genes under positive selection in different categories. The importance of phenotypic divergence coupled with chemical communication for the maintenance of this obligate mutualism highlights chemical signaling as a key mechanism of coevolution between cycads and their weevil pollinators.

## Introduction

Obligate mutualistic associations involving a high degree of specialization by both partners such as those between figs and fig wasps and yuccas and yucca moths are less common in nature, and yet the analysis of such systems is also vital to our understanding of mechanisms underlying the evolution of plant/insect interactions (Pellmyr, 2003; Herre et al., 2008). Studies of these and other pollination systems (Holland and Fleming, 1999; Kawakita, 2010) have highlighted morphological and behavioral traits associated with mutualism, and begun to identify mechanisms of diversification and speciation associated with these lineages (Althoff et al., 2012; Hembry et al., 2013; Suinyuy et al., 2015). Plant volatile production for attracting pollinators has been hypothesized as a likely mediator of specialized brood-site mutualisms (Pellmyr, 2003; Okamoto et al., 2007), yet has been largely overlooked in its contribution to diversification (Suinyuy et al., 2015). In order to fully understand the process of diversification in mutualistic, coevolving lineages, we need to learn more about the causes and consequences of species interactions. Specifically, we are interested in identifying: (1) traits driving these relationships, (2) whether these traits are diverging across closely related taxa, and (3) the role of selection in trait divergence. *Zamia* cycads and their *Rhopalotria* pollination mutualists are an ideal study system as they represent an understudied obligate brood-site pollination mutualism where the importance of volatile signaling is clear.

The ancient plant order Cycadales is an early diverging lineage of gymnosperms (Ran et al., 2018) and, unlike most other gymnosperms requires insect pollination instead of wind pollination (Terry et al., 2012). The pollination mechanism of most cycads appears to be an obligate brood site mutualism whereby highly specialized pollinators live out their lifecycle within pollen cone tissue (Mound and Terry, 2001; Terry, 2001; Terry et al., 2005, 2007). This mutualism is hypothesized to be driven by host plant volatile organic compounds (VOCs) through a push-pull pollination mechanism (Terry et al., 2007; Salzman et al., 2020). Pollinators are attracted to mid-level amounts of the host plant compound but are repelled by high levels of VOCs. Pollen and ovulate cones have a daily cycle of VOC production causing pollinators to be repulsed by the male cones and attracted to the female cones, and then attracted to the male cones once again. The pattern of plant volatile release is conserved across the Cycadales, and the behavioral response of pollinators to the daily change in expression of host plant VOC has converged between *Cycadothrips chadwicki* (Thysanoptera) and *Rhopalotria furfuracea* (Coleoptera) suggesting that this push-pull pollination mechanism is the likely pollination syndrome across the Cycadales (Salzman et al., 2020). Much of the Neotropical cycad genus *Zamia* are pollinated by *Rhopalotria* weevils and closely related genera within the Allocorynina (Tang et al., 2018). These weevils feed and reproduce on the pollen cone of their host *Zamia* (Norstog and Fawcett, 1989; Norstog et al., 1992). Conversely, the host *Zamia* species are completely dependent upon pollination services by the weevils (Norstog et al., 1986; Tang, 1987). The *Zamia—Rhopalotria* symbiosis appears to be fully mutualistic for both lineages, and the relationship has been hypothesized to exhibit phylogenetic congruence between specialized partners (Norstog et al., 1992; Stevenson et al., 1998; Tang et al., 2018). However, studies remain to be done that provide information about potential coevolutionary mechanisms involved, including those documenting reciprocal evolutionary change in each lineage (e.g., Janzen, 1980).

Chemical communication mediating the relationship between plant and pollinator has been described for one species pair, Mexican *Z. furfuracea/R. furfuracea*, and is hypothesized to be driving relationships across the two lineages (Salzman et al., 2020), but we do not know whether the plant/pollinator behavior is shared across other *Zamia/Rhopalotria* pairs, whether volatile production and perception traits are diverging in the lineages, or whether any of these traits are driven by positive selection. Here we describe the chemical communication that underlies a distantly related species pair, the Caribbean *Z. integrifolia*—*R. slossoni*. We then ask if there has been evolution of specificity in volatile perception in these two *Rhopalotria* species. Finally, we investigate phenotypic and genotypic evolution across the Caribbean *Zamia* clade to look for evidence of rapid evolution and positive selection associated with volatile production. By analyzing reciprocal evolutionary change in volatile production and insect perception, we provide investigation of coevolution in what is arguably the earliest example of an insect mediated “pollination syndrome” (Cai et al., 2018; Salzman et al., 2020).

## Materials and Methods

### Study System and Design

*Rhopalotria* and *Zamia* form a tight obligate mutualism. *Rhopalotria* and their close relatives are found across much of *Zamia* diversity where they provide necessary pollination services to their respective host *Zamia* species, which in turn provide food, brood sites, and shelter. Feeding damage does not affect reproduction or potential fitness of *Zamia* as *Rhopalotria* feed only on the disposable pollen cone parenchyma tissue. This mutualism is mediated by plant volatile production (Salzman et al., 2020), a cue that is important for lifecycle completion in both lineages. The strength of the mutualism is such that pollinators go into diapause during the 10 months of the year when reproductive services are not required by the host plant (Norstog and Fawcett, 1989; Norstog et al., 1992).

Caribbean *Zamia* species are distributed across Florida, Cuba, Jamaica, the Bahamas, Cayman Islands, Dominican Republic (but not Haiti) and Puerto Rico (Calonje et al., 2020). *Rhopalotria* species have been found associated with all populations of Caribbean *Zamia* except those on Puerto Rico (Tang et al., 2018). *Zamia integrifolia* as currently circumscribed is distributed across the southeastern United States (Florida and Georgia), the Bahamas, Cayman Islands, and Cuba (Calonje et al., 2020) although these populations are not monophyletic (Calonje et al., 2019). *Rhopalotria slossoni* is distributed across southern Florida (O’Brien and Tang, 2015) where it provides pollination services to populations of *Z. integrifolia* (Tang, 1987; Norstog et al., 1992).

### Plant Volatile Analysis

To quantify the diversity of VOC profiles in the *Zamia* clade, we collected plant volatiles from dehiscing pollen cones of 10 Caribbean taxa using headspace collection methods (see Salzman et al., 2020). We quantified volatiles from 3 to 7 plants per taxa. These plants were wild collected as seeds from native populations across the Caribbean (Supplementary Figure 1) and cultivated in a common garden at Montgomery Botanical Center (MBC) in Coral Gables, Florida. All volatiles were collected between the hours of 13:30 and 16:00 (Supplementary Table 1) across 7 days in 2019. *Zamia* phenology across the phylogeny is highly varied at MBC, but most Caribbean *Zamia* are reproductive in January/February (Griffith et al., 2012). We also captured volatiles from one pollen and two ovulate cones of *Z. integrifolia* collected from Levy County, Florida at hour and a half time points from 8:00 until 20:45. MBC accession numbers for these plants are 20050825^∗^A, 20050831^∗^A, and 20050820^∗^C. Samples were eluted as described in Salzman et al. (2020) and were run on a combined Agilent technologies 6890 network gas chromotograph and 5973 mass-selective detector using a DB-5 column (J&W Scientific Inc., Folsom CA; 30 m × 0.25 mm I.D.; film thickness, 0.25 um; splitless mode) (GC-MS). Helium was the carrier gas at a constant flow rate of 0.7 ml/min. the injector temperature was 250°C, oven temperature was held at 50°C for 1 min, and then increased 10°C/min to 170°C where it was held for 5 min.

Volatile peaks were standardized and calibrated manually to include all peaks to be comparable across samples. Bag, filter, and DCM controls showed a few contaminant peaks after 15 min retention time that were removed during integration. The ChemStation Integrator was used with the following parameters: Initial area reject = 0, initial peak width = 0.300, initial threshold = 16.0, shoulder retention = off. Integration began at 3,550 min after the solvent peak and ended at 15,000 min (ChemStation software, Agilent technologies). Peak areas were calculated to percent composition (size of individual volatile compound peak/total peak area of the sample) and were treated as phenotypic traits in subsequent trait analysis.

Percent compositions of volatile peaks were analyzed for dissimilarities within and between species (Bray-Curtis dissimilarity index with 999 permutations) using the non-parametric test ANOSIM (analysis of similarities), vegan v2.3-5 package (Oksanen et al., 2019) in R version 3.6.3 (R Core Team, 2020).

### Weevil Electroantenograph Detection (EAD)

Gas Chromotrography-Electroantenograph Detection (GC-EAD) can be used to determine the physiological capability of an insect to perceive a volatile compound. We used this technique to identify volatile compounds produced by *Zamia integrifolia* that trigger responses in the antennae of *Rhopalotria slossoni.* We also tested the specificity of pollinator response using two species of *Rhopalotria* and their respective host plants.

We used headspace collection methods (see Salzman et al., 2020) to capture volatiles from receptive ovulate (Montgomery Botanical Center (MBC) accession number 20050831^∗^A) and dehiscent pollen (MBC accession numbers 20050815^∗^1 and 20050815^∗^2) cones of *Z. integrifolia*. We then used GC-EAD (see Crook et al., 2008; Salzman et al., 2020) to determine which host plant volatiles elicited responses from the antennae of *R. slossoni*. We injected 2 μl plant volatile eluate and used a GC method where the oven temperature was held at 50°C for 1 min, and then increased 10°C/min to 300°C where it was held for 5 min. The injector temperature was 280°C and the GC outlets for the EAD and FID were 300°C. For mass spec identification of individual plant volatile compounds, samples were run separately as described above for plant volatile analysis.

Eleven *R. slossoni* weevils were tested using GC-EAD against *Z. integrifolia* cone aerations. The antennally active peak was identified on the basis of its mass spectra using the universal NIST chemical library (NIST version 2.0, 2002), Kovats index (van Den Dool and Kratz, 1963; Kovats, 1965), and by comparing its retention time and mass spec with an authentic standard obtained from Sigma-Aldrich (St. Louis, MO, catalog no. M2047). The positive EAD response was tested using GC-EAD of nine weevils against a standard of methyl salicylate (Sigma-Aldrich, St. Louis, MO, catalog no. M2047). For these standard GC-EAD runs, the oven temperature was held at 50°C for 1 min, and then increased 20°C/min to 320°C, where it was held for 5 min. All other settings remained the same.

To determine the specificity of weevil perception of potential host plants, the GC-EAD physiological responses of two species of *Rhopalotria* were compared using the volatile compounds of their host and non-host *Zamia* species. The active compound identified using antennae of *R. slossoni*, methyl salicylate, differs from the active compound identified in another *Rhopalotria-Zamia* species pair involving *Zamia furfuracea*. In the latter, the plant volatile compound 1,3-octadiene was identified as the physiologically active component eliciting a response from the mutualistic weevil partner, *R. furfuracea* (Salzman et al., 2020). Thus *R. slossoni* (*n* = 2) and *R. furfuracea* (*n* = 3) were tested against a mixture of 1,3-octadiene (ChemSampco Inc. Dallas, TX, catalog no. 7015.90) and methyl salicylate (Sigma-Aldrich, St. Louis, MO, catalog no. M2047) using the same GC-EAD set up and oven settings used for the cone aeration GC-EADs.

### Weevil Behavior

**FIGURE 1:**
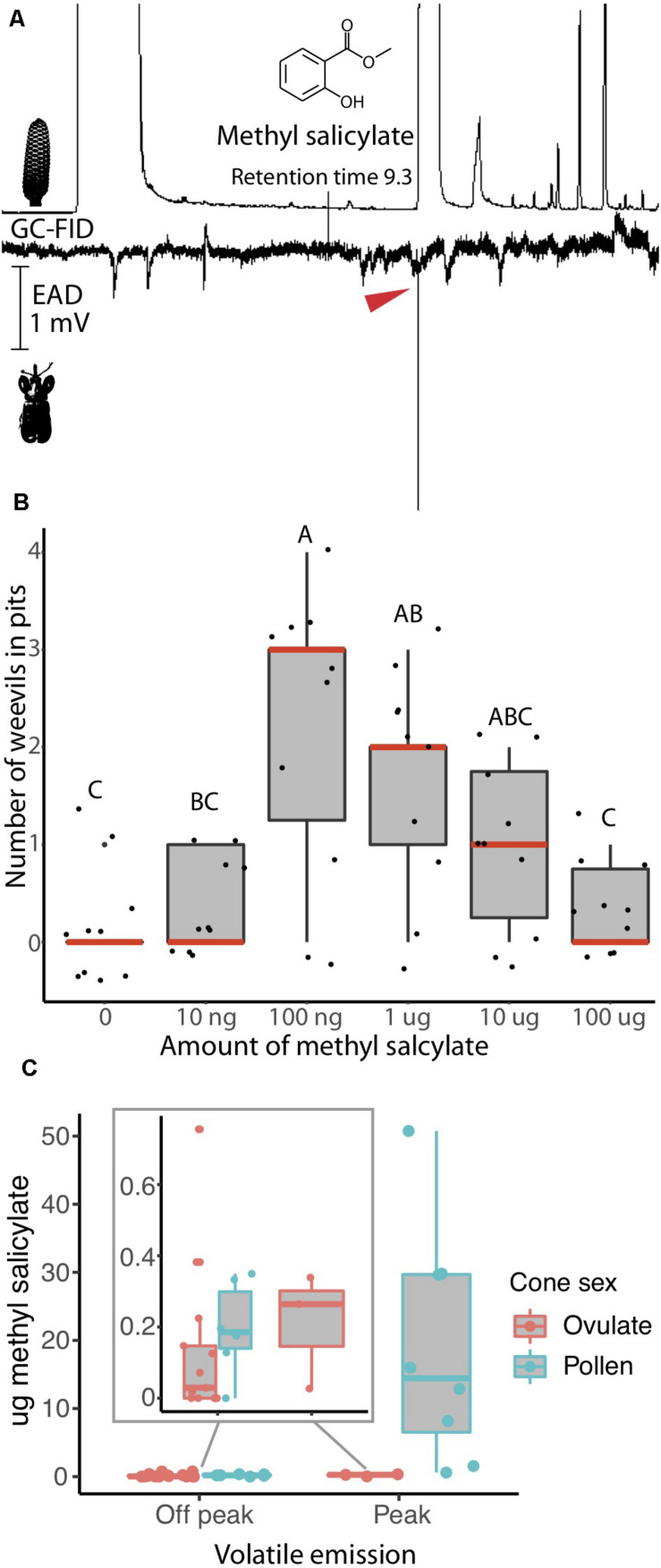
*Rhopalotria slossoni* perception and behavioral response to *Zamia integrifolia* production of methyl salicylate follows push-pull pollination (Salzman et al., 2020). **(A)** Electroantenograph detection (EAD) shows methyl salicylate elicits responses from antennae of *R. slossoni*. Plant gas chromatography flame ion detection (GC-FID) on the top, weevil electroantenograph detection (EAD) on the bottom with red arrow denoting a positive response (*n* = 11 and confirmed with a standard *n* = 9). **(B)** Pitfall test of behavior shows that methyl salicylate acts as an attractant with a non-linear response. Median values are shown in red and *p*-values are in Supplementary Table 5. **(C)** Methyl salicylate emissions from *Zamia integrifolia* pollen and ovulate cones change over the course of the day. Volatiles were sampled from 8:00 to 20:45. Peak emissions are between 13:30 and 16:00 h. Methyl salicylate production in pollen cones during off peak hours (inset) coincides with the amount most attractive to weevils in pitfall tests. Production of methyl salicylate during peak hours coincides with amounts found to be less attractive in pitfall tests. Ovulate cones also increase their emission of methyl salicylate, reaching peak attractive amounts at the same time that pollen cones become less attractive.

Using pitfall tests, we confirmed that the behavioral response of *R. slossoni* to the volatile compound methyl salicylate follows push-pull pollination in the same manner observed in *R. furfuracea* (see Salzman et al., 2020). To determine whether methyl salicylate acts as an attractant, and whether weevil attraction changes with differing amounts of the volatile compound, behavioral assays were carried out consisting of a hexane control and five dilutions of methyl salicylate in HPLC grade hexane (concentrations: 1 ng/μl, 10 ng/μl, 100 ng/μl, 1 μg/μl, 10 μg/μl) (see Salzman et al., 2020 for detailed methods). 10 μl of each dilution was used to cover the natural emission of methyl salicylate production found in *Z. integrifolia* (Figure 1C). Methyl salicylate values at peak plant expression ranging from 1 to 50 μg (Figure 1C) are expected to be repellent to *R. slossoni*. All concentrations plus the control were run simultaneously for each trial (*n* = 10). Prior to the trial, weevils were allowed to feed freely on *Z. integrifolia* cones so they would not be stressed before the trial started. Each trial was carried out by placing 4 *R. slossoni* weevils into the arena away from the pit. Arenas were closed and the entire trial was placed in the dark at room temperature (21°C) for 30 min, after which time the number of weevils in the pits were counted. Dead or copulating weevils were not counted.

As with *R. furfuracea* (Salzman et al., 2020), *R. slossoni* behavioral response to host volatile did not follow a normal distribution and therefore the raw data plus 0.5 (to account for zero values) was log transformed for statistical tests of differences in attraction to the different concentrations of methyl salicylate. Weevil attraction to different amounts of methyl salicylate was determined through an ordinary least squares model when no significant difference was found between ordinary least squares and weighted least squares models (*p* = 0.54) using the nlme package (Pinheiro et al., 2020) in R version 3.6.3 (R Core Team, 2020). *P*-values were then corrected for family-wise error using sequential Bonferroni correction.

### Morphological Trait Analysis

To quantify morphological variation across the *Zamia* clade, we photographed Caribbean *Zamia* herbarium sheets from 12 Herbaria and processed them for morphological leaflet measurements in ImageJ (version 2.0.0-rc-69/1.52n) (Rueden et al., 2017) through Fiji (Schindelin et al., 2012). Leaflet length/width were chosen as comparative morphological traits because they have been shown to be fairly consistent within and divergent between Caribbean populations (Eckenwalder, 1980). Herbarium sheets were used from A, ANS-PHILA, BNH, FTG, GH, MAPR, MICH, MO, NYBG, UPRRP, US, and USNH herbaria. We also collected leaflets from natural populations on Eleuthera, Andros Island, Grand Bahamas, New Providence and Long Island. Leaflets from the middle of the leaf rachis were selected from each herbarium sheet or individual field collection and measured for length and width. Cycad leaflets unfurl and all leaflets expand at the same time resulting in a leaflet shape that is mostly uniform across the leaf while there may be variation in size especially at the top and bottom of the leaf rachis. Size increases as the leaflet grows and fully expands. Due to the limitations of the available material and not knowing the age or developmental stage of the leaflets in herbarium material, we calculated ratios of leaflet length by leaflet width to remove any influence of size. The number of measured leaflets per population are: *Zamia* sp. (Andros Island): 34, *Z. angustifolia* (Eleuthera): 10, *Z.* sp. (Eleuthera) 48, *Z.* sp. (Grand Bahamas): 51, *Z.* sp. (New Providence): 37, *Z*. *lucayana* (Long Island): 25, *Z. integrifolia* (Florida): 19, *Z. pumila* (Puerto Rico): 12, *Z. erosa* (Puerto Rico): 6. GPS point localities were either measured in the field, copied from herbarium sheets, or manually generated using herbarium data sheet locality information and Google Maps to determine their inclusion in the studied populations. Herbarium sheets were not available for the Jamaican populations of *Z. aff. amblyphyllidia* or *Z. aff. portoricensis* and so these species were not included in the leaflet morphology analysis.

We analyzed the leaflet measurements using R version 3.6.3 (R Core Team, 2020) to determine dissimilarities (Bray-Curtis dissimilarity index with 999 permutations) using within and between populations using the non-parametric test ANOSIM (analysis of similarities) as implemented in the vegan v2.3-5 package (Oksanen et al., 2019).

### Selection in the Zamia Genome

#### Transcriptomes

We collected Plant RNA from dehiscing pollen cones of wild collected Caribbean *Zamia* seeds grown at MBC for three purposes: (1) to detect signatures of positive selection across the Caribbean clade, (2) to identify genes whose expression correlates with volatile production, and (3) to identify genes whose expression correlates with cone development. To detect signatures of selection across the Caribbean clade, microsporophyll (cone scales) were collected from dehiscing pollen cones of 10 Caribbean *Zamia* (Supplementary Table 2). To identify genes whose expression correlates with volatile production, microsporophyll cone scales were collected from *Z. integrifolia* at six time points across a day in concert with volatile headspace sampling. This sampling was done at the same time as the daily volatile collection described above using MBC accession 20050825^∗^A. Volatiles were collected for 45 min, at which point one microsporophyll cone scale was removed from the center of the cone for RNA extraction. To identify genes whose expression correlates with cone development, entire cones of *Z. furfuracea* were collected at three developmental stages: just emerging cones, young half sized cones, and immature almost full-sized cones. Two separate plants were sampled, with one cone of each stage collected on each plant.

All RNA samples were flash frozen in liquid nitrogen in the field and then stored at −80 until RNA extraction. Accession information and initial collection locality for all plants are listed in Supplementary Table 2. Cone tissue was ground to a powder in liquid nitrogen and RNA extraction was performed using the Qiagen RNeasy Plant MiniKit (Qiagen, Hilden, Germany) with the addition of 100 μl polyethylene glycol 4000 and 10 μg polyvinlypyrrolidone 40. RNA purity and integrity were evaluated using a 2100 bioanalyzer (Agilent Technologies, Santa Clara CA) using the plant RNA Nano Assay (version 1.3). Libraries were prepared from samples with a RNA Integrity Number greater than 7 using the NEBNext Ultra RNA library prep kit for Illumina (# E75305, New England Biolabs, Ipswich MA), NEBNext Poly(A) mRNA magnetic isolation module (#E7490, New England Biolabs, Ipswich MA), NEBNext multiplex oligos for Illumina (#E7335, #E7500, New England Biolabs, Ipswich MA), and Agencourt AMPure XP beads (#A63881, Beckman Coulter, Pasadena CA). Library purity and Integrity was assessed using a 2100 bioanalyzer (Agilent Technologies, Santa Clara CA) using the high sensitivity DNA Assay (version 1.03). Libraries averaged 350–500 base pairs. All 23 samples were combined in equal concentration and run across three lanes of 125 paired end reads on Illumina HiSeq2500 (San Diego CA) at Cold Spring Harbor Labs (Cold Spring Harbor NY).

Transcriptomes were filtered to remove low-quality paired-end sequence reads with Timmomatic (Bolger et al., 2014) and individually *de novo* assembled using Trinity v2.3.2 (Grabherr et al., 2011; Haas et al., 2013; Supplementary Table 3). Assembly quality was assessed using the embroyphyta lineage gene set in BUSCO v3.0.2. (Seppey et al., 2019). Through the Galaxy platform (Afgan et al., 2018; Supplementary Figure 2). Coding sequences were identified with Transdecoder v3.0.0 (Haas et al., 2013) and sequence redundancy reduced with CD-HIT-EST v4.6.4 (Li and Godzik, 2006). Orthologs were identified using the tree based ortholog identification pipeline described in Yang and Smith (2014) and briefly described here. This involved using all-by-all BlastN (Altschul et al., 1997) and Markov cluster algorithm (MCL) (Enright et al., 2002; Van Dongen and Abreu-Goodger, 2012) on all non-redundant coding sequences to obtain clusters of similar sequences. These clusters are aligned using MAFFT v7.309 (Katoh and Standley, 2013) and trimmed with Phyutility (Smith and Dunn, 2008). Cluster trees were estimated using FASTTREE v2.1.9 (Price et al., 2010). In order to account for long branches resulting from misassembly, paralogy, or recombination branches longer than 10 times the average distance to tips in its sister clade and longer than 0.4 substitutions per site were trimmed from these trees. The resulting sequence files were then realigned using MAFFT (Katoh and Standley, 2013) and used to infer gene trees with Randomized Axelerated Maximum Likelihood (RAxML) v8.2.10 (Stamatakis, 2014). These trees were subjected to an additional long branch trimming and subsetted to a taxon occupancy of 12 or more for phylogenetic analysis and 10 or more for tests of positive selection that excluded the out-group, *Zama furfuracea*, and the Caribbean *Z. lucayana* where many genes were missing due to poor transcriptome assembly. These homologous gene trees were then further pruned to a single orthologous sequence per sample using the maximum inclusion method (MI) which isolates the subtree with the highest number of taxa without taxon duplication (Dunn et al., 2008, 2013; Smith et al., 2011; Yang and Smith, 2014).

#### Plant Genomic Positive Selection

Transcriptomes of dehiscing pollen cones of ten Caribbean *Zamia* species were used to determine whether genes related to volatile production were experiencing higher rates of positive selection than other genes in the genome. The Mexican *Zamia furfuracea* was included as an outgroup. This was done in three steps briefly described here and further described below. First, genes that were found in all in-group species transcriptomes were tested for evidence positive selection. Second and separately, differential expression analysis was run on *Z. furfuracea* developmental cones to identify differentially expressed (DE) genes related to cone development and on *Z. integrifolia* cone scales collected in correlation with volatile production to identify DE genes related to volatile production. These two groups of DE genes were then used to define three gene “bins”: DE genes related to *Z. furfuracea* cone development = “reproductive development genes,” DE genes related to *Z. integrifolia* volatile production = “volatile associated genes,” and all remaining genes = “other genes.” Finally, these two data sets were brought together and the genes previously found to be experiencing positive selection were divided amongst gene “bins” to look for increased positive selection on volatile associated genes.

**FIGURE 2:**
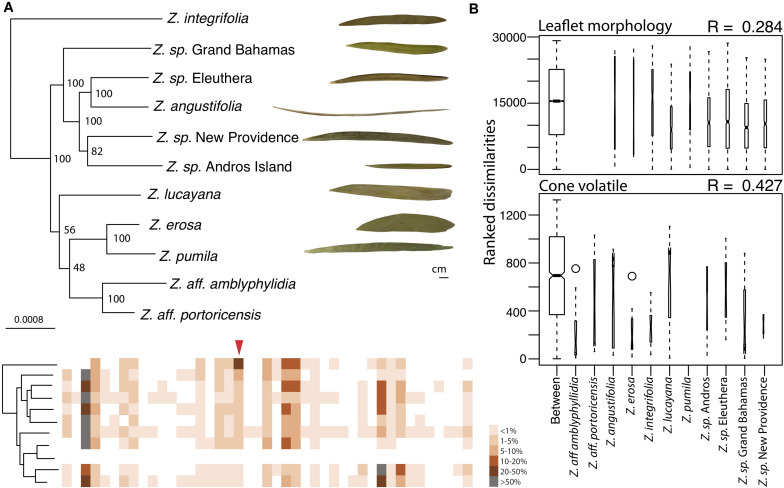
Cone volatile diversity is more different between Caribbean *Zamia* species than leaflet morphology. **(A)** Maximum likelihood phylogeny using 829 orthologous genes of Caribbean *Zamia* showing leaflet and volatile phenotypic diversity. Bootstrap values are placed at the nodes. The out-group *Z. furfuracea* has been removed from the image for clarity. Representative leaflets are included to scale for each population where living collection or herbarium records were available and a schematic of cone volatiles is provided. Individual volatile compounds are represented by boxes along the schematic and are aligned between species. Volatiles are presented as present or absent (colored or white box) and percent composition is averaged across all individuals for the species (intensity of color). Methyl salicylate is denoted with a red arrow. A close-up of these aligned volatile representations with corresponding GC-MS retention times is presented in Supplementary Figure 3, along with a dendrogram heatmap of each sample in Supplementary Figure 4. **(B)** Analysis of similarity (ANOSIM) results using leaflet shape (length/width) measurements taken from herbarium and living collections (top) and percent compositions of all volatile compounds (bottom). Boxplots of ranked dissimilarities are shown for each taxon as well as between taxa and widths are proportional to sample size. The higher the *R*-value, the more dissimilar samples are between species than within. Species without data are not included in the analysis.

First, we estimated a species tree for all Caribbean *Zamia* included in this study using 829 orthologous genes found in all 11 in-group samples and the *Zamia furfuracea* out-group. Orthologs were concatenated for a total of 797,745 aligned columns with an overall matrix occupancy of 0.96 (Statistics per sample are found in Supplementary Table 4). Models of evolution for each gene were determined using ModelFinder plus in IQ-TREE v 1.6.1 (Nguyen et al., 2015; Kalyaanamoorthy et al., 2017). All partitions were set to share the same branch lengths, with each partition allowed to have its own evolution rate. A maximum likelihood (ML) tree and bootstrap tree were then estimated together using 200 runs and random bootstrap and parsimony seeds in RAxML v8.2.10 (Stamatakis, 2014; Figure 2A).

We then investigated evidence of positive selection on the 2,487 genes that were found in 10 in-group Caribbean species using the site models (Nielsen and Yang, 1998; Yang et al., 2000) implemented in the program Phylogenetic Analysis by Maximum Likelihood v4.8 (PAML) (Yang, 2007). The species tree was pruned to remove the two taxa not used in PAML selection analysis, the out-group *Z. furfuracea* and in-group *Z. lucayana* that had a poor quality assembly as determined with BUSCO scores. Nucleotide sequences for these genes were aligned using the codon model in prank v.170427 (Löytynoja and Goldman, 2008). Individual PAML analyses for each of the 2,487 genes were run using scripts from Schultz and Sackton (2019). In tests of positive selection, ω (the ratio of non-synonymous/synonymous mutations) is determined for sites along an alignment. We tested four site models that allowed for the ω ratio to vary among codons or amino acids in the protein. These models were M1a (neutral), M2a (selection), M7 (beta), and M8 (beta and ω). We then computed likelihood ratio tests comparing likelihood scores between M1a vs. M2a and M7 vs. M8 (Nielsen and Yang, 1998; Yang et al., 2000; Wong et al., 2004) and computed *p*-values according to a χ^2^ distribution with two degrees of freedom. A false discovery rate (FDR) was applied to correct *p*-values for multiple testing using the fdrtool R package (Strimmer, 2008). 466 genes were statistically significant at 95% confidence for the preferred M2a model of selection verses the M1a neutral model and 463 genes were statistically significant for the preferred M8 model of selection verses the M7 neutral model. The 463 genes where both comparisons favored the selection model were considered to be those genes expressed in the dehiscing pollen cone and shared across Caribbean *Zamia* that are experiencing positive selection.

Separately, we used gene expression patterns to create three gene groupings: “volatile associated genes,” “reproductive development associated genes,” and “other genes.” For the “volatile associated genes,” we determined gene expression correlated with volatile production using *Z. integrifolia* samples that were collected in concert with volatile collection. For the “reproductive development associated genes” we determined gene expression correlated with pollen cone development using *Z. furfuracea* samples. For both *Z. integrifolia* volatile and *Z. furfuracea* development, expression for each individual sample was calculated using RSEM v1.2.29 (Li and Dewey, 2011) and the resulting count matrices were analyzed using the EBSeq package v1.28.0 (Leng et al., 2013; Leng and Kendziorski, 2020) in R v4.0.3 for conditional analysis. To determine gene expression pattern conditions for the “volatile associated genes,” we used the total ion abundance of methyl salicylate present in volatile samples collected in concert with *Z. integrifolia* assembled transcriptomes. Six time points from one individual were analyzed and found varying expression of methyl salicylate (Supplementary Figure 5A). However, time points one and two had similar total ion abundances (79,531 and 83,357, respectively) as did time points three and five (46,720 and 41,758, respectively). Therefore, gene expression patterns that were considered correlated with volatile production were the three in which these time point pairs, one and two and three and five, showed similar expression (Supplementary Figure 5A). Additionally, two gene expression patterns considered non-volatile associated were included, those where expression patterns were all the same or all different across all time points.

To determine gene expression pattern conditions for the “reproductive development associated gene,” we used three developmental stages of *Z. furfuracea* pollen cones: just emerged, young half sized cones and immature almost full-size cones. Gene expression patterns that were considered correlated with pollen cone growth were those that differed by developmental stage as either all stages different or the first or last stage different (Supplementary Figure 5B). The expression pattern with no difference between reproductive stages was included and considered not associated with reproductive structure growth. Gene expression conditions were determined using EBSeq as libraries were set to vary between samples and 25 iterations were run on both datasets separately until convergence was reached as determined by a difference of less than 0.001 in the hyper-parameters α and β and the mixture parameter ρ for each run. Genes were considered volatile associated if they had a greater than 0.95 posterior probability of being in one of the three volatile associated gene expression patterns (Supplementary Figure 5A). Genes were considered reproductive development associated genes if they had a posterior probability greater than 0.95 of being in one of the three reproductive stages gene expression patterns (Supplementary Figure 5B).

The volatile and reproductive associated genes were then filtered to include only those genes that were present in the 2,487 genes used in the positive selection PAML analysis. Additionally, any genes that were found in both the volatile and the reproductive associated gene bins were removed from both, generating the final “volatile associated” bin with 146 genes and “reproductive development associated” bin with 674 genes. The “other” gene bin includes all remaining 1,667 genes not found in either the “volatile associated” and “reproductive development associated” gene bins as well as those genes that were found to overlap. Finally, we determined the distribution of positive selection in Caribbean *Zamia* pollen cone transcriptomes across gene bins by blasting the 463 genes found to be experiencing positive selection against the three gene bins (Supplementary Figure 5C). The 30 positively selected genes in the “volatile” bin were annotated using Trinnotate v3.2.1 (Bryant et al., 2017). The 21 identified gene ontology molecular functions (Ashburner et al., 2000; Gene Ontology Consortium, 2021) were then used to categorize genes into 7 functional categories (Supplementary Figure 6).

## Results

We investigate the coevolution of the *Rhopalotria* and *Zamia* lineages by determining that the mechanism of interaction is consistent across two species pairs and provide evidence of reciprocal evolution in traits related to this mechanism in both lineages. Using insect physiology and behavior, we demonstrate that plant-insect chemical communication and push-pull pollination is indeed consistent across these two species pairs and is driving the relationship of the *Rhopalotria/Zamia* mutualism. We then show that these traits are diverging in both lineages and that there appear to be higher rates of positive selection associated with volatile production.

### Chemical Communication Is a Mechanism of Mutualism Across Rhopalotria and Zamia Species Pairs

We identify the chemical communication underlying the *R. slossoni/Z. integrifolia* mutualism and find the push-pull mechanism to match that demonstrated in *R. furfuracea/Z. furfuracea* (Salzman et al., 2020) in which weevils have specialized to perceive only the signal emitted by their respective host plant. To do this, we first identified plant volatile compounds that are physiologically perceived by the pollinator in the *R. slossoni/Z. integrifolia* species pair. Using electroantenograph detection (EAD) we find that *R. slossoni* is physiologically capable of perceiving only one compound emitted by its host plant, and we identified this as methyl salicylate (Figure 1A), a compound previously reported from *Z. integrifolia* (Pellmyr et al., 1991).

In order to begin to determine whether chemical communication and push-pull pollination is indeed the mechanism of interaction across *Rhopalotria/Zamia* lineages, we compared the volatile behavioral response and plant volatile production in *R. slossoni/Z. integrifolia* with those identified in an earlier study of *Rhopalotria/Zamia* mutualism. Using the same pitfall tests we used in the earlier work, we found that *R. slossoni* are attracted to methyl salicylate but that the response is non-linear (Figure 1B). Weevils are significantly more attracted to mid-level amounts of methyl salicylate than to low or high amounts (*p*-values in Supplementary Table 5). To determine if methyl salicylate could be performing the push-pull pollination function in *Z. integrifolia*, we analyzed the production of methyl salicylate across the day. We found that methyl salicylate production does undergo a large increase in the pollen cone (Figure 1C).

To test whether insect perception of host plant compounds may be diverging across these lineages, we performed a reciprocal test of volatile perception between *R. slossoni* and *R. furfuracea*, an obligate pollinator of *Z. furfuracea* previously found to perceive and respond to the *Z. furfuracea* compound 1,3-octadiene (Salzman et al., 2020). Using mixtures of methyl salicylate and 1,3-octadiene, we find that weevils show a positive electroantenographic response to the compound produced by their host plant, but that neither weevil responds to the electroantennally active compound of the other.

### Zamia Volatile Phenotypes Are More Diverged Than Morphological Phenotypes

We compared volatile and morphological divergence across the Caribbean *Zamia* clade to look for evidence of trait evolution related to the mutualism in the plant lineage and we found a greater level of dissimilarity between Caribbean *Zamia* cone volatiles as compared to leaflet morphology (Figure 2). Our analysis uncovered 48 volatile compounds across the entire Caribbean clade with an average of 15 compounds per individual, ranging from 9 to 26. *Zamia integrifolia* had the lowest average number of VOCs per species at 12.67 and *Z. lucayana* had the highest average at 18.14. For leaflet morphology, ratios of leaflet length by width (L/W) range from 4.232 at the widest to 68.508 at the thinnest, with an average of 15.51. *Zamia angustifolia* had the thinnest leaflets with an average L/W ratio of 49.5 and *Z. erosa* had the widest leaflets with an average ratio of 6.70, with all others falling within 9.5 (*Z. lucayana*) to 19.0 (*Z. integrifolia*).

We used an analysis of similarity to determine if volatile phenotypes are more dissimilar between taxa than leaflet morphology. The ANOSIM *R*-value compares the mean of ranked dissimilarities between groups to the mean within groups. Values below 0 indicated dissimilarities are greater within groups than between groups, whereas those close to 1.0 suggest dissimilarity between groups and those close to 0 indicate that the distribution of values is even between and within groups. Our analysis showed a low level of dissimilarity between Caribbean *Zamia* leaflet morphology using Bray-Curtis distance (*R* = 0.284) with a significance of 0.001. Conversely, we found an *R*-value closer to 1, and therefore, more dissimilar between groups for plant volatile profiles (*R* = 0.4268, Bray-Curtis distance) with a significance of 0.001.

### Searching for Selection on Zamia Volatile Associated Genes

To investigate the role of selection on divergence in plant volatile traits, we sequenced *de novo* transcriptomes of pollen dehiscing cones of Caribbean *Zamia* and looked for evidence of positive selection in three gene “bins” determined by the differential expression pattern of genes compared to methyl salicylate production or cone developmental stage. Transcriptome assembly overall was of high quality with BUSCO scores for 11 of the 12 species all showing 70.3–84.6% complete BUSCO genes (Supplementary Figure 2). The single poor quality assembly was *Zamia lucayana* with only 8.6% complete BUSCO genes, so we included this species in the phylogeny but not in subsequent selection analysis. The maximum likelihood score for the phylogeny was −1,237,542.0107. The remaining 10 in-group Caribbean species transcriptomes had 2,487 orthologous genes, of which we measured 463 (18.6%) experiencing positive selection. Our differential expression analysis and filtering identified 146 “volatile associated” genes, 674 “reproductive development associated” genes, and 1,667 “other” genes among the 2,487 orthologs. The ratio of positively selected genes among the “volatile associated” genes was 30 out of 146 (20.6%), among the “reproductive development associated” genes was 144 out of 674 (21.4%), and among “other” genes was 289 out of 1,667 (17.3%) (Supplementary Figure 5). Although it is tempting to interpret this as showing a trend for more of the volatile or reproductive development associated genes to be under positive selection than other genes, the difference is not significant with a proportion test (*p* = 0.1373). and our initial hypothesis was not supported by these data. Gene annotation of the 30 positively selected genes in the “volatile” bin described 21 molecular functions that fell into 7 categories. The highest percentage was uncategorized genes (41.46%, *n* = 17), followed by enzymatic activity (24.39%, *n* = 10) and nucleotide binding (12.19%, *n* = 5) genes. The remaining categories are metal ion binding (9.76%, *n* = 4) transcription (4.88%, *n* = 2), ATP associated (4.88%, *n* = 2), and transport (2.44%, *n* = 1) genes (Supplementary Figure 6).

## Discussion

Our results provide evidence in support of co-evolutionary reciprocal evolution in the *Rhopalotria-Zamia* pollination mutualism. The relationship between two distantly related species pairs of *Rhopalotria* pollinators and their *Zamia* cycad hosts is characterized by a highly specific chemical communication that is potentially evolving under positive selection in the host plants, with phenotypes related to volatile production in the plants and perception in the insects displaying significant differences. We first identified a key trait underlying the relationship and confirmed that chemical communication mediated the interaction by showing that the physiological and behavioral responses of *R. slossoni* to a primary compound in the volatiles expressed by their host plant (Figure 1) mirror the push-pull pollination mechanism previously described (Terry et al., 2007; Salzman et al., 2020). The behavioral response to methyl salicylate matches the behavioral response of *R. furfuracea* to its host plant compound 1,3-octadiene (Salzman et al., 2020). In the *R. furfuracea/Z. furfuracea* pollination system, *Z. furfuracea* initiates pollination by instigating the movement of *R. furfuracea* out of the host pollen cone through cyclical increases in the amount of 1,3-octadiene (Salzman et al., 2020). Here we found that methyl salicylate production undergoes a large increase in the pollen cone (Figure 1C) similarly to the pattern observed with 1,3-octadiene in *Z. furfuracea* (Salzman et al., 2020). This burst in production of methyl salicylate occurs in the mid-afternoon and coincides with the time that *R. slossoni* are most active (Tang, 1987). We infer from these results that that chemical communication and push-pull pollination is the usual mechanism underlying *Rhopalotria* and *Zamia* associations and is likely to be found throughout the Cycadales (Salzman et al., 2020). We then determined that these traits are diverging between closely related taxa in both lineages. We find that *Rhopalotria* pollinators respond to different chemicals in the volatiles released by their respective host plants, and do not respond to an inappropriately matched chemical; they appear to be physiologically uncapable of perceiving their non-host plant. Plant volatiles differ more between species than their leaf morphology does (Figure 2), suggesting an increase in diversification in volatile phenotype compared to other morphological phenotypes, although without additional data about trait evolution within and between these taxa of *Zamia*, it is difficult to conclude much about these relative differences.

Disentangling the contribution of mutualistic species interactions from the myriad of influences on diversification remains elusive (Maron et al., 2019), yet fine scale investigations of obligate mutualisms requiring a high degree of specialization between both partners offer some of the best potential insights into the role of coevolution in diversification. The yucca/yucca moth system is the only brood site pollination mutualism where these questions have been explicitly addressed so far, and research has shown that pollinating sister species of *Tegeticula* moths are driving divergence in floral traits related to pollination (Godsoe et al., 2008) and contributing to reproductive isolation (Smith et al., 2009) in two varieties of *Yucca brevifolia*. Our data suggest that chemical communication is likely to play a central role in lineage diversification for both parties involved here as well. Selection can drastically alter plant volatile profiles over the course of just a few generations (Zu et al., 2016; Gervasi and Schiestl, 2017; Ramos and Schiestl, 2020) and the striking differences in volatile production and perception shown here suggest that these traits are essential in securing species specificity.

Volatile variation across populations has been described in two cycad species, *Encephalartos ghellinckii* (Suinyuy and Johnson, 2018) and *E. villosus* (Suinyuy et al., 2013, 2015). The former species has diverged in pollinator assemblage between populations while the later has not, but pollinators do show preferences for volatiles from their host populations both physiologically and behaviorally. Here, we show that two species of *Rhopalotria* weevils are physiologically specialized to perceive the active component of only their own host plant VOCs, thereby exhibiting adaptation to their respective host plants. Scanning electron micrograph images of the antennae of different species of *Rhopalotria* show high variation in the distribution and density of sensory pockets (O’Brien and Tang, 2015) that are likely to correspond with the physiological and behavioral differences described here. Together, the evidence is consistent with population divergence in volatile production or perception likely acting as an inhibitor to gene flow and thereby facilitating diversification. We therefore looked for evidence of selection operating on genes related to volatile emission. Although a fifth of these genes were under positive selection, we found no significant differences between the proportion of genes under selection in different categories (Supplementary Figure 5). It is possible that this was due to the limitation of sample size of gene bins or because our process of binning volatile vs. reproductively related gene expression was not sufficient to capture relevant differences. Overall, while we were unable to confirm positive selection on volatile associated genes, our data nevertheless identify significant differences in volatile production and perception in these interactions, and point toward additional research that could be done to investigate how these interacting lineages may be affecting each other’s evolutionary trajectories.

Controversy still exists regarding the potential evolutionary trajectory of obligately coevolved mutualisms such as the push-pull pollinations of cycads (e.g., Vamosi et al., 2014; Day et al., 2016) and with further research, this system is likely to provide important insights. Divergence in chemical communication traits in both lineages has clear potential for contributing to lineage diversification, and while the fossil record supports a scenario of plant coevolution with insect pollinators occurring before the rise of angiosperms (Crepet, 1979) and even specifically involving cycads (Cai et al., 2018), the *Rhopalotria/Zamia* mutualism is likely to be much younger than these associations. *Zamia* is thought to have arisen ∼ 85 Ma (Condamine et al., 2015; Calonje et al., 2019), about the same time as the earliest potential divergence time for *Rhopalotria* (McKenna et al., 2009). However, crown diversification of *Zamia* occurred much more recently at 22–9 Ma, with a rapid radiation occurring within the last ∼5 Ma (Calonje et al., 2019).

To date, phylogenetic relationships between species of *Rhopalotria* have been based on a cladistic analysis of 89 morphological, behavioral, and host characters, together with 300 base pairs of 16s (Tang et al., 2018). This analysis described one species in Florida, *Rhopalotria slossoni*, one species, *R. dimidiata*, in the Bahamas and Cayman Islands, and one in Jamaica, *R. meerowi*, with relationships between them unresolved. However, our initial analysis of 650 base pairs of CO1 (unpublished) suggests greater diversity within these populations than has been previously appreciated. Larger scale phylogenetic analysis is needed to determine the diversity of *Rhopalotria* and related cycad pollinating members of the tribe Allocorynina in order to assess the potential co-diversification history of the two lineages. The interaction with specialized cycad herbivores on the evolutionary trajectory of plant volatiles may also be important. The interactive effects of pollinator attraction and herbivory can have major and rapid effects on the evolution of plant traits (Ramos and Schiestl, 2019, 2020). Methyl salicylate is involved in reproduction for *Zamia integrifolia* yet likely also has other ecological functions as it is known to play a great many roles in insect behavior from attraction (James, 2003) to deterrence (Ninkovic et al., 2003) and even as an anti-aphrodisiac transferred in nuptial gifts (Andersson et al., 2000). Larvae of the lycaenid butterfly, *Eumaeus atala* (Lepidoptera) larvae in Florida and the Caribbean are major pests of Zamia (Whitaker and Salzman, 2020) and while the chemical ecology of host plant localization or response to methyl salicylate remains unknown in *E. atala*, it likely further impacts the evolution and diversification of both *Zamia* and *Rhoplaotria* lineages.

## Data Availability Statement

The sequencing data has been deposited into a publicly accessible repository (link: https://dataverse.harvard.edu/dataverse/CaribbeanZamia).

## Author Contributions

SS, MC, DS, NP, and RH conceived of the project. SS performed all field, laboratory and behavioral experiments, GCMS, and statistical analysis. DC performed EAD analysis. MC collected specimens in the field for morphological analysis. All authors contributed to the article and approved the submitted version.

## Conflict of Interest

The authors declare that the research was conducted in the absence of any commercial or financial relationships that could be construed as a potential conflict of interest.

## Supplementary Material

Supplementary Figure 1 Map of Caribbean Zamia cultivated at Montgomery Botanical Center.

Supplementary Figure 2 BUSCO genes recovered in assembled transcriptomes.

Supplementary Figure 3 Volatile diversity across the Caribbean *Zamia* clade.

Supplementary Figure 4 Heatmap and dendrogram of Caribbean *Zamia* volatiles.

Supplementary Figure 5 Caribbean *Zamia* gene expression and positive selection.

Supplementary Figure 6 Functional analysis of volatile associated genes experiencing positive selection.

Supplementary Table 1 Accession information on Caribbean *Zamia* used in volatile analysis.

Supplementary Table 2 Accession information on Caribbean *Zamia* used for RNA.

Supplementary Table 3 Summary of transcriptome assemblies.

Supplementary Table 4 Matrix occupancy stats for phylogenetic reconstruction.

Supplementary Table 5 Sequential Bonferroni corrected *p*-values for pitfall test.
