# Supplementary material for: Cycad-Weevil Pollination Symbiosis Is Characterized by Rapidly Evolving and Highly Specific Plant-Insect Chemical Communication
Source: Front Plant Sci. 2021 Apr 30;12:639368. doi: 10.3389/fpls.2021.639368 (PMC8121082; doi:10.3389/fpls.2021.639368)
Supplement: Supplementary Figure 1 — Map of Caribbean Zamia cultivated at Montgomery Botanical Center. [file Data_Sheet_1.docx]

Supplementary Material

# Tables

Table S1: Caribbean *Zamia* plants used in diversity volatile analysis for phenotypic traits. Plants were wild collected as seeds and cultivated in the common garden at Montgomery Botanical Center.

| Species | Collection locality | Accession | Collection time | % Dehisced |
| --- | --- | --- | --- | --- |
| *Z. erosa* | Puerto Rico | 20030278*A | 14:39 | 50 |
| *Z. erosa* | Puerto Rico | 20030279*C | 14:40 | 50 |
| *Z. erosa* | Puerto Rico | 20030277*C | 14:41 | 50 |
| *Z. erosa* | Puerto Rico | 20030281*A | 14:42 | 75 |
| *Z. erosa* | Puerto Rico | 20030278*B | 14:21 | 25 |
| *Z. lucayana* | Long Island | 20090822*P60 | 15:46 | 80 |
| *Z. lucayana* | Long Island | 20090819*B | 16:01 | 75 |
| *Z. lucayana* | Long Island | 20090817*A | 16:04 | 75 |
| *Z. lucayana* | Long Island | 20090812*C | 16:06 | 50 |
| *Z. lucayana* | Long Island | 20090824*E | 14:25 | 25 |
| *Z. lucayana* | Long Island | 20090826*E | 14:26 | 25 |
| *Z. lucayana* | Long Island | 20090823*F | 14:27 | 25 |
| *Z. angustifolia* | Eleuthera | 20110148*B | 15:51 | 50 |
| *Z. angustifolia* | Eleuthera | 20100183*J | 15:54 | 25 |
| *Z. angustifolia* | Eleuthera | 20110154*A | 15:55 | 55 |
| *Z. angustifolia* | Eleuthera | 20100184*R | 15:57 | 25 |
| *Z. angustifolia* | Eleuthera | 20110153 | 15:47 | 50 |
| *Z. angustifolia* | Eleuthera | 20110148 | 15:50 | 20 |
| *Z. integrifolia* | Putnam county, Florida | 20050839*G | 14:23 | 75 |
| *Z. integrifolia* | Levy county, Florida | 20050776*A | 14:25 | 75 |
| *Z. integrifolia* | Volusia county, Florida | 20050882*D | 14:30 | 25 |
| *Z. integrifolia* | Flagler, Florida | 20050870*D | 14:14 | 85 |
| *Z. integrifolia* | St. Augustine, Florida | 93808*X | 14:18 | 50 |
| *Z. integrifolia* | St. Augustine, Florida | 93808*CC | 14:20 | 50 |
| *Z. sp. grand bahamas* | Grand Bahamas | 20110161 | 16:11 | 25 |
| *Z. sp. grand bahamas* | Grand Bahamas | 20110162 | 16:11 | 75 |
| *Z. sp. grand bahamas* | Grand Bahamas | 20110162*G | 14:30 | 25 |
| *Z. sp. grand bahamas* | Grand Bahamas | 20110160 | 15:50 | 85 |
| *Z. sp. grand bahamas* | Grand Bahamas | 20110162*E | 14:31 | 50 |
| *Z. sp. grand bahamas* | Grand Bahamas | 20110162-2 | 15:50 | 75 |
| *Z. sp. new providence* | New Providence | 20100148 | 16:09 | 25 |
| *Z. sp. new providence* | New Providence | 20100148-2 | 16:09 | 85 |
| *Z. sp. new providence* | New Providence | 20090809*B | 16:10 | 25 |
| *Z. sp. eleuthera* | Eleuthera | 20110140 | 16:10 | 25 |
| *Z. sp. eleuthera* | Eleuthera | 20110145*B | 14:45 | 75 |
| *Z. sp. eleuthera* | Eleuthera | 20110144*E | 14:45 | 50 |
| *Z. sp. eleuthera* | Eleuthera | 20100185*G | 16:09 | 25 |
| *Z. sp. eleuthera* | Eleuthera | 20110146 | 15:44 | 25 |
| *Z. sp. andros* | Andros Island | 2010114 | 16:12 | 75 |
| *Z. sp. andros* | Andros Island | 20100148 | 14:39 | 80 |
| *Z. sp. andros* | Andros Island | 20100118 | 15:46 | 10 |
| *Z. aff. portoricensis* | Jamaica | 20080216*B | 14:37 | 35 |
| *Z. aff. portoricensis* | Jamaica | 20080217*D | 14:38 | 50 |
| *Z. aff. portoricensis* | Jamaica | 20080218*E | 14:19 | 85 |
| *Z. aff. portoricensis* | Jamaica | 20010216*G | 14:16 | 85 |
| *Z. aff. portoricensis* | Jamaica | 20080219 | 15:44 | 10 |
| *Z. aff. amblyphylidia* | Jamaica | 20080207*QQ | 14:47 | 75 |
| *Z. aff. amblyphylidia* | Jamaica | 20080207*WW | 14:46 | 25 |
| *Z. aff. amblyphylidia* | Jamaica | 20080207*CC | 14:46 | 50 |
| *Z. aff. amblyphylidia* | Jamaica | 20080207*R | 16:14 | 75 |
| *Z. aff. amblyphylidia* | Jamaica | 20080207*K | 16:18 | 75 |
| *Z. aff. amblyphylidia* | Jamaica | 20080207*G | 16:16 | 25 |

Table S2: *Zamia* plants used for transcriptome assembly of pollen dehiscing cones. *Z. integrifolia* cone scales were collected at ~1.5 hour intervals in concert with volatile collection. *Z. furfuracea* cones were collected whole at three developmental stages from the same plant; just emerging, young (reaching half of fully developed cone height), and immature (reaching almost full height with no pollen sac development).

| Species | Collection locality | Accession |
| --- | --- | --- |
| *Z. angustifolia* | Eleuthera | 20110152 |
| *Z. erosa* | Puerto Rico | 20030281*A |
| *Z. pumila* | Puerto Rico | 20080285*D |
| *Z. lucayana* | Long Island | 20020875*J |
| *Z. aff. portoricensis* | Jamaica | 80221 |
| *Z. aff. amblyphyllidia* | Jamaica | 20080207*G |
| *Z. integrifolia* | Levy County, Florida | 20050825*A |
| *Z. sp. grand bahamas* | Grand Bahamas | 76402*A |
| *Z. sp. andros island* | Andros Island | 200100114 |
| *Z. sp. new providence* | New Providence | 200100148 |
| *Z. furfuracea* | Mexico | 93418L |
| *Z. furfuracea* | Mexico | 93418C |

Table S3: Summary of transcriptome assemblies

| Taxa | Number genes | N50 length | Median length | Average length | Assembled bases | CD-HIT clusters |
| --- | --- | --- | --- | --- | --- | --- |
| *Z. amblyphyllida* | 92,244 | 1560 | 469 | 880.81 | 81,249,776 | 37,822 |
| *Z. angustifolia* | 110,965 | 1282 | 393 | 735.59 | 81,624,342 | 36,128 |
| *Z. erosa* | 91,409 | 1598 | 477 | 893.51 | 81,674,431 | 34,739 |
| *Z. furfuracea* | 188,004 | 1266 | 344 | 683.38 | 128,477,518 | 52,392 |
| *Z. integrifolia* | 190,372 | 1330 | 381 | 742.13 | 141,280,461 | 67,331 |
| *Z. lucyana* | 28,650 | 550 | 348 | 490.83 | 14,062,379 | 10,709 |
| *Z. aff. protorcensis* | 70,317 | 1507 | 545 | 909.09 | 63,924,581 | 26,830 |
| *Z. pumila* | 158,434 | 1170 | 375 | 697.37 | 110,486,343 | 49,715 |
| *A. sp. andros* | 85,710 | 1657 | 499 | 931.56 | 79,843,780 | 34,424 |
| *Z. sp. eleuthera* | 100,432 | 1453 | 436 | 823.24 | 82,679,932 | 37,703 |
| *Z. sp. grand bahamas* | 97,856 | 1471 | 436 | 830.09 | 81,229,016 | 37,170 |
| *Z. sp. new providence* | 85,121 | 1653 | 499 | 930.89 | 79,238,115 | 33,693 |

Table S4: Matrix occupancy stats for phylogenetic reconstruction using 829 genes with a total of 797,745 aligned columns and an overall matrix occupancy of 95.5%.

| Species | Total Characters | Percent Orthologs | Percent Characters |
| --- | --- | --- | --- |
| *Z. amblyphyllida* | 785,946 | 100 | 98.5 |
| *Z. angustifolia* | 780,436 | 100 | 97.8 |
| *Z. erosa* | 790,376 | 100 | 99.1 |
| *Z. furfuracea* | 781,976 | 100 | 98.0 |
| *Z. integrifolia* | 780,493 | 100 | 97.8 |
| *Z. lucyana* | 510,197 | 100 | 64.0 |
| *Z. aff. protorcensis* | 785,524 | 100 | 98.5 |
| *Z. pumila* | 787,741 | 100 | 98.8 |
| *A. sp. andros* | 787,013 | 100 | 98.7 |
| *Z. sp. eleuthera* | 782,607 | 100 | 98.1 |
| *Z. sp. grand bahamas* | 782,461 | 100 | 98.1 |
| *Z. sp. new providence* | 785,243 | 100 | 98.4 |

Table S5: Sequential Bonferroni corrected p-values for pit fall test.

|  | 10 ng | 100 ng | 1 ug | 10 ug | 100 ug |
| --- | --- | --- | --- | --- | --- |
| 0 | 1.0000 | 0.0010 | 0.0090 | 0.1591 | 1.0000 |
| 10 ng |  | 0.0092 | 0.0647 | 0.5902 | 1.0000 |
| 100 ng |  |  | 1.0000 | 0.4535 | 0.0032 |
| 1 ug |  |  |  | 1.0000 | 0.0242 |
| 10 ug |  |  |  |  | 0.3502 |

Figure S1: Map of Caribbean Zamia cultivated at Montgomery Botanical Center (MBC). Plants used in cone volatile analysis are denoted with their species names. These plants were grown from seed wild collected at these locations and grown in the conservation garden at MBC in Miami Florida.

Figure S2: BUSCO genes recovered in assembled transcriptomes

Figure S3: Volatile diversity across the Caribbean *Zamia* clade. Each column represents all of the GC-MS peaks present in the pollen dehiscing cone volatiles of one Caribbean taxon. Each row represents the GC-MS retention time of the volatile compound. The average percent composition of the volatile compound in each taxon is represented by the color of intensity. No color indicates that the compound is not present in that taxon. Methyl salicylate is denoted with a red arrow. The antennal active compounds are unknown in all species other than *Z. integrifolia*, but may be those with high expression.

Figure S4: Heatmap and dendrogram of Caribbean *Zamia* volatiles created using the base R function heatmap(). Volatiles are organized by retention time and analyzed by percent composition. Plant samples are clustered by similarity. The darker the red, the higher the percent composition of that volatile compound for that sample. Methyl salicylate is denoted with a red arrow.

Figure S5: Caribbean *Zamia* genes correlated with volatile production appear to show a slight trend in experiencing greater ratios of positive selection than other genes in the genome; however, this difference is not significant and we cannot conclude that a larger proportion of these genes are under positive selection. A.) Gene expression patterns used to determine volatile associated genes. The boxed letters describe the three possible gene expression patterns that could account for gene expression correlated with volatile production. Transcriptomes were collected concurrently with volatiles and total ion abundance of methyl salicylate was used to determine gene bins. There are two cases where volatile production could be considered similar, those in red and those in green. Different letters in a pattern indicate that gene expression was allowed to vary between those transcriptomes. The repeat of a color and letter in the pattern box indicates that gene expression was required to be the same in that pattern. Only genes that fit into one of the three patterns can be considered volatile associated. B.) Gene expression patterns used to determine reproductive development associated genes. Transcriptomes were generated from three time points of pollen cone development. Genes whose expression pattern fell into one of three patterns where gene expression difference was between subsequent developmental stages were included in the bin. C.) The proportion of genes in the volatile associated bin appear to be experiencing higher ratios of positive selection than other orthologs, but the difference is not significant.

Figure S6: Functional analysis of the 30 genes that are positively selected in the volatile associated bin as determined by gene ontology searches.
